# What do we know about the needs and challenges of health systems? A scoping review of the international literature

**DOI:** 10.1186/s12913-017-2585-5

**Published:** 2017-09-08

**Authors:** Federico Roncarolo, Antoine Boivin, Jean-Louis Denis, Rejean Hébert, Pascale Lehoux

**Affiliations:** 1 Institute of Public Health Research of Université de Montréal (IRSPUM), Montreal, Canada; 2 Department of Family Medicine, Faculty of Medicine, Université de Montréal, Montreal, Canada; 3 Research Center of the Université de Montréal Health Center (CRCHUM), Montreal, Canada; 4 Canada Research Chair in Patient and Public Partnership, Montreal, Canada; 5 Department of Health Management, Evaluation and Policy, School of Public Health, Université de Montréal, P.O. Box 6128, Branch Centre-ville, Montreal, QC H3C 3J7 Canada; 6 Canada Research Chair in Governance and Transformation of Health Organizations and Systems, Montreal, Canada; 7 Université de Montréal Chair on Responsible Innovation in Health, Montreal, Canada

**Keywords:** Health system strengthening, Challenges, Health service delivery, Human resources, Governance, Leadership, Health innovation

## Abstract

**Background:**

While there is an extensive literature on Health System (HS) strengthening and on the performance of specific HSs, there are few exhaustive syntheses of the challenges HSs are facing worldwide. This paper reports the findings of a scoping review aiming to classify the challenges of HSs investigated in the scientific literature. Specifically, it determines the kind of research conducted on HS challenges, where it was performed, in which health sectors and on which populations. It also identifies the types of challenge described the most and how they varied across countries.

**Methods:**

We searched 8 databases to identify scientific papers published in English, French and Italian between January 2000 and April 2016 that addressed HS needs and challenges. The challenges reported in the articles were classified using van Olmen et al.’s dynamic HS framework. Countries were classified using the Human Development Index (HDI). Our analyses relied on descriptive statistics and qualitative content analysis.

**Results:**

292 articles were included in our scoping review. 33.6% of these articles were empirical studies and 60.1% were specific to countries falling within the very high HDI category, in particular the United States. The most frequently researched sectors were mental health (41%), infectious diseases (12%) and primary care (11%). The most frequently studied target populations included elderly people (23%), people living in remote or poor areas (21%), visible or ethnic minorities (15%), and children and adolescents (15%). The most frequently reported challenges related to human resources (22%), leadership and governance (21%) and health service delivery (24%). While health service delivery challenges were more often examined in countries within the very high HDI category, human resources challenges attracted more attention within the low HDI category.

**Conclusions:**

This scoping review provides a quantitative description of the available evidence on HS challenges and a qualitative exploration of the dynamic relationships that HS components entertain. While health services research is increasingly concerned about the way HSs can adopt innovations, little is known about the system-level challenges that innovations should address in the first place. Within this perspective, four key lessons are drawn as well as three knowledge gaps.

**Electronic supplementary material:**

The online version of this article (10.1186/s12913-017-2585-5) contains supplementary material, which is available to authorized users.

## Background

Research on health systems has been rapidly growing all over the world and, with methods that have become more rigorous, it has gained in clarity. According to the World Health Organization (WHO), a health system (HS) “consists of all organizations, people and actions whose primary intent is to promote, restore or maintain health” [1]. Because they include “efforts to influence determinants of health,” HSs are “more than the pyramid of publicly owned facilities that deliver personal health services” and integrate actions carried outside professional health sector [1]. For example, HSs encompass:

*a mother caring for a sick child at home; private providers; behaviour change programmes; vector-control campaigns; health insurance organizations; occupational health and safety legislation. It includes inter-sectoral action by health staff, for example, encouraging the ministry of education to promote female education, a well-known determinant of better health* [1]*.*

In the past decades, scholarship on HSs sought to improve our understanding of their key components and examined more closely the role played by various actors and strategies to improve their performance and increase their responsiveness to emerging population health needs [1–7]. Health services research has also increasingly paid attention to the way HSs are supposed to adapt to medical advances and adopt technological innovations. This scholarship has at times produced recommendations to facilitate the uptake of new technologies (e.g., computerized medical records, telehealth) that could strengthen specific HSs, but not much work has sought to take stock, globally, of the needs and challenges of HSs that innovations should address in priority. Furthermore, since HS research may examine the quality of service delivery in specific clinical areas, the needs of specific patient groups or the outcomes of specific care providers, it is published in a variety of journals. As a result, evidence regarding the needs and challenges of HSs is scattered across multiple scientific journals, in a format that remains not easily accessible to policymakers and to those engaged in the development of health innovations.

In this paper, we thus report the findings of a scoping review whose aim was to take stock of, and classify the needs and challenges of HSs that have been investigated in the peer-reviewed scientific literature since 2000. We defined “challenges” as the emerging and enduring problems that destabilize the current functioning, performance or sustainability of HSs. “Needs” refer to human, financial or material resources that are deemed necessary to improve and sustain its functioning.

A scoping review, because of its inclusiveness, is an appropriate method to navigate and circumscribe what is known about a complex and multifaceted research topic [8]. As explained below, we used the framework developed by van Olmen and colleagues [7] to address the following research questions: What kind of research has been conducted on HS challenges? Where has this research been conducted, in which health sectors and on which populations? What types of challenge have been documented? To what extent do the reported challenges vary across countries? Ultimately, having a better grasp on this international literature could help identify key priorities for HS improvement and knowledge gaps that call for scholarly attention.

### Conceptual framework

**Fig. 1:**
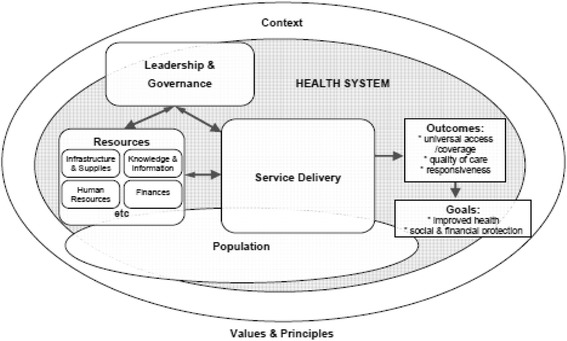
The dynamic health system framework. Note: Reproduced with permission from: van Olmen et al. in [9].

The current literature on HSs mobilizes different conceptual models and typologies, with some success in their application to describe HS components, but also with certain shortcomings. One of the critical points is defining what falls within HSs and what falls beyond them, i.e., external factors [3]. For the purpose of our scoping review, we needed a conceptual framework that could help us to classify different types of HS challenge, without oversimplifying the issues at play.^1^ The “dynamic health system” framework elaborated by van Olmen and colleagues proved particularly well suited [9]. It was conceptualized using the WHO building blocks as a starting point, but it articulates more clearly the interactions and dynamic equilibriums among these building blocks. More specifically, van Olmen’s framework consists of: 1) leadership and governance; 2) resources, which include infrastructure and supplies, human resources, knowledge and information, and finances; 3) service delivery; 4) population; 5) context; 6) values and principles; 7) outcomes; and 8) goals (see Fig.1). This framework recognizes that HSs are shaped by context and history and evolve as open systems: their components constantly and dynamically interact.

Because our intent was to take stock of the international scholarship on HSs, we also needed a classification system for the geographical areas on which focused the articles. Rather than classifying countries according to their level of income or their geographic position, we grouped them along the 2015 Human Development Index (HDI), which was developed by Mahbub ul Haq and Amartya Sen and supported by the United Nations Development Programme [10]. HDI combines indicators that are highly relevant to population health: life expectancy at birth, mean years of schooling and expected years of schooling, and gross national income per capita. Examining the extent to which the challenges reported in the international literature vary across countries along the HDI could better reflect the ability of HSs to address population health needs.

## Methods

Scoping reviews are particularly well suited for taking stock of existing knowledge and identifying important research gaps. Scoping reviews differ from systematic reviews in that they do not seek to assess the quality of the available evidence [8]. According to Arksey and O’Malley [11], scoping reviews aim to map rapidly the key concepts underpinning a research area as well as the main sources and types of evidence available. More specifically, we followed the scoping review steps described by Levac et al. [12].^2^

### Search strategy: Identification of databases and relevant articles

We sought to strike a balance between breadth and comprehensiveness, and developed our bibliographic search strategy in two steps: firstly, we identified a set of articles (*n* = 43) that represented good examples of the papers we were looking for, that is peer-reviewed articles that examined the challenges, problems, priorities or needs of HSs. These articles were identified through consultation with experts, a structured search on Pubmed and a hand search of the references cited in the articles that we had used to prepare our scoping review. This initial set of studies helped us to formulate our search strategies more explicitly (see Additional file 1). A scientific library specialist was hired to refine and execute our search strategy.

In order to capture articles that belonged to a range of disciplines relevant to HS research, a total of 8 peer-reviewed online databases were searched in March 2016: Pubmed, EMBASE, PsycINFO, International Bibliography of the Social Sciences-ProQuest (IBSS), Sociological abstracts-ProQuest, Worldwide political science abstracts-ProQuest, Public affairs Information Service-ProQuest (P.A.I.S.) and Web of Science-SCI, SCII. All database searches were limited to languages spoken by the team members (English, French and Italian) and a publication date between January 2000 and April 2016. To reduce the occurrence of citations that would be excluded, articles without an abstract were excluded automatically or with a post query when the database system did not do it automatically.

### Article selection process

After exclusion of duplicates, two reviewers independently screened 4820 abstracts against our inclusion and exclusion criteria. To be included in our review the papers had to: 1) have an abstract; 2) be written in English, French or Italian^3^; and 3) present HS challenges or needs (see search terms in Additional file 1). Articles describing specific vertical programs or technologies were excluded. Reviewers met regularly during the review process to discuss any uncertainty regarding the selection criteria and to refine them. If the relevance of a study was unclear from the abstract, then the full article was retrieved. Disagreements were resolved through discussions with the research team. In line with the methodology of scoping reviews, the final selection was not made according to the quality of the studies [11]. Because abstracts cannot be assumed to be representative of the full article or to capture its full scope [13], the articles that met the inclusion criteria were read by the first author to ascertain whether they properly addressed our research questions.

### Data collection and charting

A data chart was built and refined with the research team to determine which specific variables to document for the analyses. To this end, an Excel file with variables falling into four broad domains was created: 1) citation of the article (authors, title, year, affiliation of the 1st author); 2) details regarding the nature and scope of the article (type of article, objectives, country studied, respondents, area of interest, target population); 3) HS challenges addressed in the article (as categorized below); and 4) excerpts describing the key challenges.

Several research team meetings were carried out during the data extraction period and the data charting form was updated and modified iteratively.

### Collating, summarizing and reporting the results

In order to take stock of what is known about HS challenges, our analytical strategy was to combine descriptive statistics (i.e., distribution of frequencies) and qualitative content analysis. We extracted for each single article all the HS challenges that had been examined and used a single category to classify each challenge. The goal was to be able to report and illustrate the challenges as closely as possible as the authors had described them, while relying on our analytical framework to maintain consistency. These categories were operationalized as explained below.

#### Human resources

In van Olmen and colleagues’ framework, this category refers to the individuals whose actions are meant primarily to protect and improve health. Human resources consist in health service providers, health managers and support workers who operate in private and public organizations [1]. Challenges in this category include the availability of the health workforce considering that health workers should be available where and when needed in terms of number, geographical distribution and skill-mix. Availability is thus modulated by training capacity, recruitment policies and workforce distribution strategies. Effective health workers should be competent and master the technical knowledge and skills required to provide care of high quality, but also possess the interpersonal skills needed to engage in patient-centred and professional care. Challenges include, for instance, lack of commitment or excessive workload [9].

#### Finances

HSs should raise adequate funds to ensure that citizens can use services when needed and are protected from catastrophic expenditures and impoverishment that may follow from having to pay for these services themselves [14]. Financing entails the acquisition, allocation and pooling of financial resources to contribute effectively to the desired goals and outcomes [7]. The way in which HSs are funded affects the delivery of services and is affected by the governance of HSs [7]. Challenges in this category include HS financing, increasing costs, financial unsustainability and lack of financial autonomy.

#### Infrastructure and supplies

This category refers to the ‘hardware’ of HSs. To be efficient and support proper care delivery, infrastructure should be accessible by potential users, well equipped, well maintained and adapted to population needs. A reliable supply system for technologies, drugs and other commodities is essential to the functioning of HSs [7]. Challenges in this category may entail poor availability and quality, unaffordability, and procurement and storage issues.

#### Knowledge and information

A variety of health information systems may contribute to the production, analysis, dissemination and use of reliable and timely health information by decision-makers and practitioners at different levels of the HS, both on a regular basis and in emergencies [1].

#### Leadership and governance

This category refers to the government’s role in health policy and its relations with actors whose activities impact population health. Challenges include overseeing and steering the whole HS, i.e., its private and public entities, while protecting the public’s interest. Leadership and governance entail both political and technical actions since competing demands for limited resources need to be reconciled, especially in shifting environments. For example, health policymakers have to respond to rising patients’ expectations, more pluralistic societies, reforms or a shifting private sector. Effective leadership and governance seek to develop strategic policy frameworks, which may entail effective oversight, regulation, accountability, transparent funding mechanisms and patient and community empowerment [1, 9].

#### Service delivery

This category refers to the various packages of care and services delivered for the prevention, promotion and treatment of acute and chronic conditions. Challenges include access to services (affordability, acceptability and geographical accessibility), comprehensiveness of services (e.g., from rehabilitation to palliative care), the vertical integration and coordination of health services and referral systems, continuity of care, quality and efficiency of services. This category thus refers to the immediate outputs of the resource inputs (see Fig. 1).

#### Context and population

HSs are affected by contextual and demographic factors, which call for responsiveness and adaptation to social, economic, technological, cultural, political, regulatory and environmental changes and transitions over time. Populations cannot be viewed as simple ‘targets’ or ‘beneficiaries.’ History, culture and traditions shape over time the way individuals and groups perceive and respond to diseases. For instance, stigma may be attached to certain conditions, impeding proper care to be sought and delivered. Challenges in this category include long-standing trends such as aging, migration and particular mixes of chronic and infectious diseases as well as sporadic events such as epidemics, conflicts, wars and environmental disasters.

#### Principles and values

This last category encompasses the ethical challenges associated to all of the above described categories such as inequalities in access and the sociocultural dimensions that underpin appropriate service delivery. For instance, certain services need to be tailored to the religious beliefs of specific patient groups and care providers.

#### Locating countries along the HDI

When applicable, the HSs described in the articles were classified along the 2015 HDI [15]. For instance, an article examining HSs in Burundi, Liberia, Mozambique, Rwanda, Sierra Leone and Uganda was classified in the low HDI category since all these countries belong to the same HDI group. In contrast, an article examining Tanzania, Mexico and the United States (U.S.) could not be classified along the HDI and was thus categorized as “other.” This last category also included papers that addressed global challenges or referred to countries, like Kosovo, whose HDI score was not available. Statistical significance across HDI groups was tested using Chi Square.

## Results

### What kind of research has been conducted on HS challenges since 2000?

**Fig. 2:**
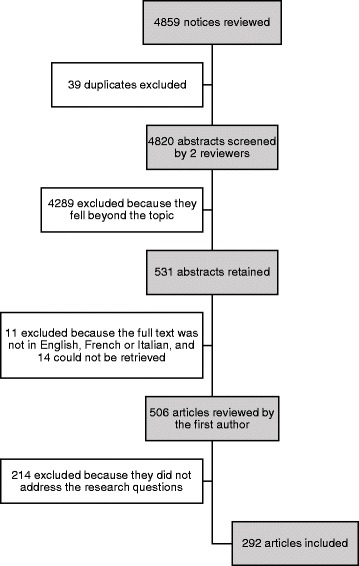
Flow diagram of the articles selection process

As Fig. 2 indicates, after exclusion of duplicates and the independent screening of 4820 abstracts by two reviewers, a total of 531 abstracts initially met our inclusion criteria. The full text could not be retrieved for 14 of these references and 11 were excluded because the articles were not written in English, French or Italian. A total of 506 articles were read by the first author to ascertain whether they addressed our research questions and, ultimately, 292 articles were formally included in our scoping review.

**Fig. 3:**
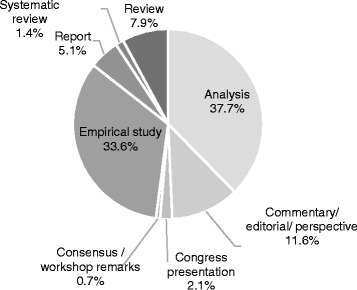
Characteristics of the articles (*n* = 292) included in our scoping review

As Fig. 3 indicates, four of these articles were systematic reviews [16–19], a third were formal empirical studies (33.6%) and a little more than a third (37.7%) were classified as ‘analyses’ since they did not rely on primary research. Among the empirical studies, the majority were qualitative (63.2%), while 13.1% of them applied a mixed method approach. Scholarly interest in HS challenges steadily increased since 2000; the number of articles published per year was higher from 2012 to 2015, with a peak of 36 articles in 2013. Overall, most of the articles examined challenges at a national or federal level (65.8%).

### Where has this research been conducted, in which health sectors and on which populations?

Among the 38 articles that could not be classified along the HDI were papers addressing the whole world (39.5%), the European Union (10.5%), low and middle income countries (7.9%) and Sub-Saharan African countries (7.9%). While the majority of the articles that could be classified along the HDI studied challenges in countries falling within the very high HDI category (60.1%), it is worth underscoring that up to 28.3% of them addressed HSs in the U.S. Table 1 shows the distribution of the countries accounting for 5% and more of all articles that could be classified within one of the four HDI categories. As Table 1 indicates, in each HDI group there is at least one country that is over-represented when compared to the others: the U.S. in the very high HDI category (47.7%); China in the high HDI category (34.8%); India and South Africa in the middle HDI category (28.6% and 25%); and Pakistan in the low HDI category (20.7%).

**Table 1:** Distribution of country-specific articles along the HDI: only countries accounting for 5% and more of all papers in their HDI category are shown

HDI category	Country	n.	%
Low HDI	Pakistan	6	20.7
	Nigeria	3	10.3
	Papua and New Guinea	3	10.3
	Ethiopia	2	6.9
	Kenya	2	6.9
	Malawi	2	6.9
	Uganda	2	6.9
	All articles	29	100
Medium HDI	India	8	28.6
	South Africa	7	25.0
	Zambia	3	10.7
	Bangladesh	2	7.1
	Ghana	2	7.1
	All articles	28	100
High HDI	China	16	34.8
	Malaysia	4	8.7
	Brazil	3	6.5
	Iran	3	6.5
	All articles	46	100
Very high HDI	United States	72	47.7
	Canada	18	11.9
	Australia	17	11.3
	All articles	151	100

A total of 99 countries were reported. Countries with a proportion of articles below 5% within their HDI category included: Low HDI: Afghanistan; Burundi, Liberia, Mozambique, Rwanda, Sierra Leone and Uganda; Ivory Coast; Lesotho; Myanmar; Sierra Leone; Somalia; Tanzania; Zimbabwe. Medium HDI: Cambodia; Iraq; Kyrgyzstan; Syria; Tajikistan; Vietnam. High HDI: Armenia; Mexico; Russia; Sri Lanka; Belarus; Brazil and Colombia; Georgia; Jamaica; Jordan; Macedonia; Mongolia; Oman; Romania; Serbia; Thailand; Tunisia. Very high HDI: United Kingdom; Belgium; Croatia; Hungary; Israel; Italy; Japan; Korea; Lithuania; Sweden; United Arab Emirates; Argentina; Australia and United Kingdom; Austria, Belgium, England, Germany, Italy, The Netherlands, Portugal and Spain; Chile; Cyprus; Estonia; European Union (15 countries); Finland; Germany; Greece; Iceland; New Zealand; Norway; Poland; Portugal; Saudi Arabia; Singapore; Slovakia; Spain; Western Europe and Switzerland

A total of 175 papers (59.9%) did not address any specific health sector, but referred to the entire HS. The remaining 117 papers (40.1%) focused on one health sector or more. Table 2 shows the distribution of the sectors (*n* = 128) examined in this sub-group of articles. The most frequently researched sectors included mental health, which accounted for 25.8% of the sectors identified and infectious diseases (mostly HIV), which accounted for 11.7%, primary care (10.9%) and non-communicable diseases (9.4%).

**Table 2:** Distribution of sector-specific articles

Health sectors	n.	%
Acute care	1	0.8
Addiction	1	0.8
Cancer care	7	5.5
Community clinics	3	2.3
Dental health	7	5.5
Disaster medicine	1	0.8
Home care and long-term care	6	4.7
Infectious diseases	15	11.7
Maternal and infant health	10	7.8
Mental health	33	25.8
Non-communicable diseases	12	9.4
Palliative and end of life care	3	2.3
Pediatrics	2	1.6
Primary healthcare	14	10.9
Public health	9	7.0
Sexual and reproductive health	3	2.3
Rehabilitation	1	0.8
Total	128	100

Since the articles could address more than one health sector, the sum of health sectors (n = 128) is higher than the number of sector-specific articles (*n* = 117)

While 214 papers (73.3%) reported challenges for the population as a whole, 78 papers (26.7%) addressed challenges specific to one target population or more. For instance, an article addressing the elderly people of a Chinese community living in a Western country had two target populations: elderly people and a minority. Table 3 shows the distribution of the populations (*n* = 98) targeted by this sub-group of articles. The most frequently studied target populations included elderly people (18.4%), people living in remote or poor areas (16.3%), visible or ethnic minorities (15.3%), and children and adolescents (15.3%).

**Table 3:** Distribution of population-specific articles

Target populations	n.	%
Children and adolescents	15	15.3
Elderly people	18	18.4
Immigrants and refugees	9	9.2
Low socioeconomic status populations	7	7.1
People with chronic diseases	3	3.1
Groups with specific health issues	11	11.2
Poor urban, rural and remote areas	16	16.3
Post war and disaster areas	2	2.0
Visible and ethnic minorities	15	15.3
Women	2	2.0
Total	98	100

Since the articles could refer to more than one target population, the number of populations (n = 98) is higher than the number of population-specific articles (*n* = 78)

To summarise, the majority of the available evidence on HS challenges relates to countries with a very high HDI score, does not focus on a specific health sector and reports challenges for the population in general. A large part of this evidence is specific to HSs in the U.S.

### What types of HS challenge have been documented by investigators?

**Fig. 4:**
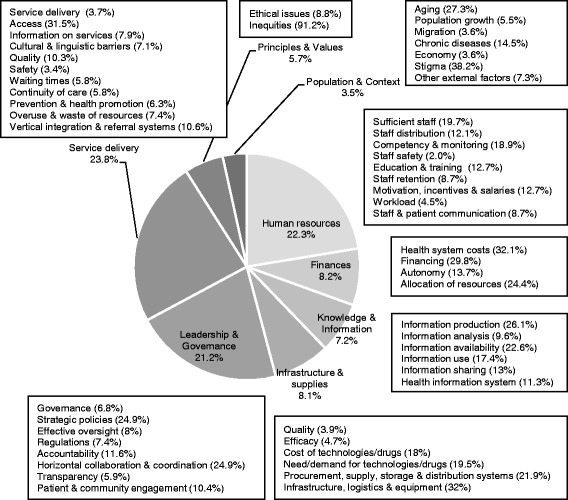
Distribution of the challenges (*n* = 1590) reported in the articles

Drawing on the categories derived from van Olmen and colleagues’ framework, Fig. 4 shows the distribution of the challenges reported in the whole set of papers. The challenges that have been most documented fall in three categories: health service delivery (23.8%), human resources (22.3%) and leadership and governance (21.2%). We discuss below the specific challenges we identified within each category; their distribution is also indicated in Fig. 4.

#### Human resources

Human resources challenges, which accounted for 22.3% of all challenges reported, mostly referred to the number of staff available (19.7%), its competency (18.9%) and its distribution (12.1%). Challenges in staff distribution could refer to particular areas within a country (rural, remote) or to the types of setting (public or private) wherein service delivery took place. For instance, the following quote describes how mental health workforce availability and distribution are connected to one another:

*Overall, human resources are not sufficient and are unequally distributed in the different geographical areas of [Brazil]. The number of psychiatric nurses is insufficient in all geographical areas of the country. Psychologists outnumber psychiatrists in all regions of the country. The distribution of human resources between urban and rural areas is also disproportionate* [20]*.*

Our corpus of articles equally underscored issues related to health workforce education and training (12.7%), and to staff motivation, incentives and salaries (12.7%).

Challenges that were less frequently examined included communication between health workers and patients (8.7%), health workforce retention (8.7%), workload (4.5%) and staff safety (2.0%). One typical challenge surrounding health workforce retention, “brain drain,” was described as a phenomenon happening both across and within countries:

*The low level of 0.9 physicians per 1000 population has seriously constrained the capacity in meeting the population’s needs in Malaysia. The movement of doctors to private hospitals further exacerbated the manpower shortage in public hospitals. Consequently, one-third of the total number of physicians who remain in the public sector struggle to provide services for two-thirds of the total number of hospital beds in the country* [21]*.*

#### Finances

Finances challenges accounted for only 8.2% of all challenges. Within this category, investigators mostly emphasized the rising costs of HSs (32.1%) and problems in securing financial resources for health (29.8%). Despite the fact that financing challenges accounted for a small percentage of the overall challenges documented in the literature, they were often framed as threat to sustainability:

*The most serious issue in Japan’s current healthcare system is the management of rising national health expenditure associated with the aging of the population. Unless appropriate countermeasures are taken, national healthcare expenditure is projected to increase from 33 trillion yen in 2006 to 56 trillion yen —a 1.7-fold increase— 19 years later in 2025* [22]*.*

Challenges raised by the allocation of resources (e.g., across private and public sectors, different HS’s domains or rural and urban areas) accounted for 24.4% and the lack of financial autonomy for 13.7%.

#### Infrastructure and supplies

Challenges related to infrastructure and supplies accounted for the 8.1% of all challenges. In this category, inadequate infrastructure, problems in logistics and the lack of adequate equipment predominated (32%). It was followed by issues in procurement, distribution and storage of supplies (21.9%), the difficulty of dealing with the rising demand for technologies and drugs (19.5%) and with their growing costs (18%).

As we will further discuss in the next section, challenges in this category varied in nature and intensity not only across rich and poor countries, but also across geographical regions within the same country. The following quote, describing infrastructure and supplies challenges in South Africa, offers a more generic perspective:

*Despite considerable investment in public infrastructure, poorer, rural areas [in South Africa] generally have more frequent stock-outs of key medical supplies, less access to diagnostic test facilities, emergency transport and various clinical equipment, and less basic infrastructure* [23]*.*

Other challenges documented in this category included efficacy and quality of supplies, accounting respectively for 4.7% and 3.9%.

#### Knowledge and information

Challenges in this category represented only 7.2% of all challenges. The production of information (26.1%) and the availability of data (22.6%) were the most frequently reported challenges. Issues in data collection could refer to data related to the patient or to service provision. For instance, for Ben Romdhane and colleagues, *“health information [in Tunisia], whether relating to patient information or service organization, was at best patchy and data could not be linked”* [24]*.*

The need to increase information use (17.4%) alongside knowledge sharing and translation (13%) was underscored, highlighting how such evidence could strengthen HSs:

*Even as research discoveries inform evidence-based practices, implementation remains suboptimal, and consequently, major gaps between evidence and practice persist. Large studies of healthcare delivery show that only about half (55%) of U.S. citizens receive necessary care; and, fewer than half of physician practices incorporate recommended processes of care. This is not a failure of knowledge; it is a failure to create a process by which new knowledge is applied and incorporated into daily practices* [25]*.*

Evidence-based clinical practice was reported as a means to address such shortcomings:

*Although there have been some significant advancements, Australia still lags behind the UK and New Zealand in having a comprehensive approach to improving chronic disease management in general practice. This is in part due to the lack of systematic based guidelines through audit and incentives* [26]*.*

The obstacles surrounding the implementation of health information systems (11.3%) and the need to build capacity in information analysis (9.6%) were less documented.

#### Leadership and governance

Within this broad category, which accounted for 21.2% of all challenges, the most frequently reported issues included strategic policies (24.9%) and horizontal cooperation (24.9%), followed by accountability (11.6%) and patient and community engagement (10.4%). For instance, the transition from pediatric to adult services of young people with mental health needs was described as a strategic planning issue:

*Although lack of fiscal resources often impedes development of services for young adults, the main impediments are separation of child and adult mental HSs, a lack of leadership and a lack of prioritisation of this age group, often the result of disconnected commissioning structures whereby services for children and young people, which are often relatively small in size, ‘lose out’ against a larger and more numerous range of services for adults* [17]*.*

The horizontal collaboration challenges we categorized had to do with inter-sectoral coordination. For instance, improving communication and cooperation with non-health sectors such as transportation and education was underscored. Horizontal collaboration challenges were described as being inseparable from those related to inter-organizational cooperation, for instance, collaboration between privately- and publicly-run services. Reflecting on the “poor systemic coordination” and the “significant functional overlap with services provided by private clinics and the government health center,” Cristofalo et al. underscored that health managers in the U.S*.: “felt that collaboration between clinics and outside agencies was poor, citing difficulty contacting providers in extra-mural mental health organizations, poor follow-up and communication* [27]*.”*

Accountability challenges were reported when, for instance, decentralization policies made clearer the gaps and overlaps between regional and national purviews: *“the conflict of competency between the region and the local health units [in Italy] is still much debated and remains unsolved*” [28]. Accountability challenges were described as characterizing either the central-level or district-level authorities:

*District governments have had little experience with such responsibilities in the past and although it makes perfectly good sense to encourage local decision-making in relation to priority-setting and resource utilization, limited capacity – for governance, planning and implementation and evaluation of programmes – raise serious issue. Pakistan’s health system fails to hold individuals and organizations accountable for their actions* [29]*.*

The adequate implementation of strategies to empower patients and communities was described as a crucial issue in HS strengthening. One of the arguments was that such empowerment could help identify needs that matter the most:

*Stakeholders [in Japan] agree on the movement toward more community-oriented mental health, but questions remain on how, how fast, and how far to take the reform measures. […] Family and consumers should be involved from the beginning of the planning process, not because their involvement is politically correct but because only they know the real needs* [30]*.*

Another argument for engaging the community was to be able to replicate positive results in other domains of care:

*We have trained voluntary treatment partners for [Directly Observed therapy, Short Course for tuberculosis in Papua and New Guinea] and the model has produced good success... A similar approach may be successful with [antiretroviral therapy]* [31]*.*

Other challenges documented in this category referred to transparency (5.9%), effective oversight (8%), regulations (7.4%) and governance (6.8%).

#### Service delivery

Service delivery challenges accounted for 23.8% of all challenges. By far, the challenge that has been reported the most is access (31.5%), followed by the need to improve vertical integration and referral systems (10.6%) and quality (10.3%).

Access challenges were defined in terms of affordability, acceptability and geographical features. These distinctions will be further examined in the next section because they reveal both commonalities and variations within and across HDI categories. In general, affordability challenges were described as follows:

*Financial barriers to access also influence health service use [in South Africa]. Increases in perceived payments difficulties for people with low income may reflect the costs of transport or worsening household economic conditions. There are also specific concerns about affordability problems at the hospital level. The rapid cost spiral in medical schemes has continued unabated since the 1980s and has made insurance increasingly unaffordable* [23]*.*

Problems in vertical coordination of care were examined within and across levels of care. For instance:

*Several [African] countries’ health systems have a weak organisational structure, which leads to uncoordinated activities at all levels of care. The collapse of primary and secondary health facilities has put serious pressure on tertiary health facilities that are not optimally prepared* [32]*.*

Issues pertaining to referral systems were classified alongside vertical coordination issues since they typically underscored the lack of effective coordination mechanisms between different care providers: *“the coordination of primary and secondary care as well as of acute and long-term care suffers from fragmented responsibilities” in Austria* [33]*.*

Several articles pinpointed variations in quality of services: *“major gaps in the quality and reliability of [US Veteran] healthcare persist”* [25]. Quality issues as perceived by patients were also reported: *“Patients reported that perceived quality problems such as excessive wait times or difficulty reaching providers by phone felt disrespectful and therefore unethical”* [34].

Challenges that were moderately represented in the service delivery category included information on services (7.9%), overuse and waste of resources (7.4%), cultural and linguistic barriers (7.1%), and prevention and health promotion (6.3%).

Finally, challenges that were less often reported referred to waiting times (5.8%), continuity of care (5.8%), service delivery (3.7%) and safety (3.4%). For instance, issues in continuity of care occurred when catering to vulnerable populations.

*Discharge planning for homeless patients is difficult [in the U.S.]. Homeless patients were said to be medically stable to leave the hospital, but still in need of basic medical care that is not available in shelters or on the street. Other participants said that hospital discharge may be delayed if an appropriate place is not available, and length of hospital stay would be longer. Hospital staff members are obligated to discharge patients who are medically stable, but shelter staff are unable to provide medical care for recuperation* [35]*.*

#### Context and population

Despite the fact that adapting HSs to shifting economic, social, political, cultural and demographic contexts has long been on the health services and policy research agenda, the context and population category accounted for only 3.5% of all challenges. Within this category, one of the sociocultural challenges most frequently reported was stigma (38.2%). This challenge was associated to service delivery in particular areas, such as sexually transmitted diseases, mental health, physical handicap or women’s health. For instance, lack of knowledge and awareness among healthcare providers about the health issues women with disabilities face in the U.S. was underscored:

*[Women] reported that healthcare providers often defined women with disabilities solely in terms of their disabling condition. Women said they often felt depersonalized by and burdensome to healthcare providers. They described encountering negative judgments about their sexual and reproductive choices; they found it difficult to advocate for their children with providers who did not support their decisions to have children. Women frequently reported that providers did not suggest pelvic examinations or mammograms and that if the women pursued such screening, they encountered difficulty finding experienced providers and accessible facilities* [36]*.*

The necessity of dealing with an aging population (27.3%) and the growing prevalence of chronic diseases (14.5%) was often mentioned, but these challenges remained framed as unmanageable factors having consequences over the financial sustainability of HSs:

*The aging of the population not only increases national healthcare expenditures, but also puts Japan’s universal medical insurance system on the verge of crisis and collapse* [22]*.*

Likewise, external political and environmental factors (7.3%) such as natural catastrophes, terroristic attacks or warfare, were reported as sporadic uncontrollable events that affect HSs. Issues less often reported included population growth (5.5%), migration (3.6%) and economic dynamics (3.6%).

#### Principles and values

This last category accounted for 5.7% of all challenges. Inequalities dominated by far (91.2%), which may be seen as culminating from the challenges that affect other areas of HSs. For instance, Phua et al. described inequalities as a phenomenon that interlaced economic growth, health service delivery coordination and governance:

*In Transitional economies (*e.g.*, China, Mongolia, Vietnam, Laos, Cambodia) with the collapse of central planning and the lack of new redistributive mechanisms for health care provision and finances, uneven development between geographical areas and disparities among population groups are highly accentuated. In newly industrialized economies there are widening disparities in resource utilization and the quality of care between the public and the private sectors* [37]*.*

Specific ethical issues were less frequently documented (8.8%) and often referred to priority-setting or improving the quality of end-of-life care, which was described as a core ethical challenge for US Veterans’ medical centers [34]. To rank the most important ethical challenges plaguing HSs, Breslin et al. gathered the views of Canadian experts on a variety of challenges such as informed consent, surrogate decision-making, end of life decisions, etc. For the managers they interviewed, knowing *“how to establish acceptable limits on the provision of care to individual patients within a population-based framework”* posed an ethical challenge and *“clinicians struggled when determining how and when limiting care to patients was ethically defensible”* [38].

To summarise, the types of challenge that were reported the most within the set of articles we reviewed fall in three categories: health service delivery, human resources, and leadership and governance.

### To what extent do the challenges reported in the scientific literature vary across countries?

Countries from the south face HS challenges that profoundly differ from those experienced in northern countries. For Jacobs and El-Sadr, the challenge of ensuring health equity *“is far greater in the global south, with health systems in many low- and middle- income countries in deep crisis.”* According to these authors:

*In these countries, health systems are plagued by inadequate human, financial and infrastructural resources, poor governance, weak leadership and management and lack of service delivery models appropriate for specific health threats and burden of disease* [39]*.*

**Fig. 5:**
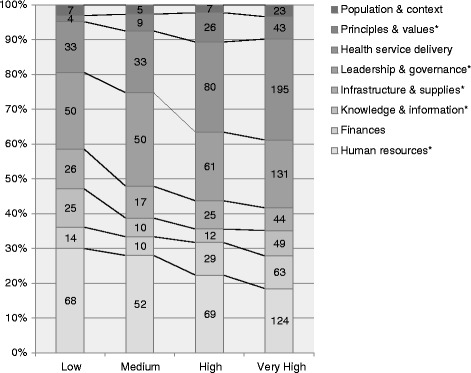
Distribution of the challenges (*n* = 1590) within HDI categories. Note: Statistical significant differences (*p* < 0.05) were found in the number of challenges across HDI for the five categories marked with an *. Articles that could not be classified along the HDI were excluded.

Our scoping review supports a closer examination of the distribution of the challenges that have been reported in the international scientific literature since 2000 within each HDI category, which is illustrated in Fig. 5. This figure shows that across the four HDI categories a roughly comparable level of attention has been given to six types of challenge. This holds for the categories of challenge that were less often reported, such as population and context, principles and values, infrastructure and supplies, knowledge and information, and finances, as well as for the leadership and governance category, which accounted for 21.2% of all the challenges reported. For the remaining two categories, Fig. 5 shows that health service delivery challenges were more frequently reported in the very high HDI countries than in the low HDI countries. The reverse is observed for human resources challenges. We thus further explore these two findings below.

#### Health service delivery challenges along the HDI

In proportion to the number of the articles published, attention given to health service delivery challenges is more important in very high HDI countries that in low HDI countries (ranging from 29% to 15%). This may reflect the growing specialization in medical care that comes with an increased supply of services in wealthier countries. It also illustrates some of the dynamic equilibriums that characterize the relationships between the various components of HSs, as emphasized by van Olmen and colleagues [7].

For example, in rich countries, over-prescription, over-hospitalisation and waste of resources have been associated with the increased use of new, expensive technologies, which of course bear direct implications on finances:

*Rampant and unnecessary utilisation of high-tech equipment and procedures abounds without due consideration for cost-effectiveness, efficacy and safety. In the developed economies, problems of rising costs due to the excessive consumption of high-tech medical care are persistent, especially when bills are paid by generous health care finances systems that offer incentives for providers to over-service* [37]*.*

While new technology may not in itself be problematic [40], it is the need to define and manage what a proper use of technology entails that raise specific health service delivery issues:

*Health technology is also considered a major cause of increased health spending, largely due to its improper use; its development and diffusion [in Spain] leads to an increase in indications for inappropriate medical and surgical procedures, unnecessary pharmaceutical prescriptions, or an increase in the population targeted for treatment* [41]*.*

In countries within the low HDI category, the proper use of new drugs and technologies was typically associated to the challenges affecting infrastructure and supplies, which vary from one country to another. For example, in South eastern Nigeria:

*The facilities surveyed in most cases are adequate in terms of size and layout, but usually grossly inadequate in terms of furnishings and utilities. Most buildings are dilapidated and need renovations. […] All the primary health centres have stock-out of drugs. The secondary however have all basic essential drugs like chloroquine, ampicillin, anaesthetics,* etc. *Most of the equipment are old and non-functional and need replacement. Maintenance culture is poor* [42]*.*

In countries within the high HDI category, poor governance and strategic planning were described as inducing waste of resources, like in Armenia: *“there is an overcapacity of beds and staff in psychiatric hospitals, leading to the unnecessary admission of chronic patients who would be more appropriately treated in a community setting”* [43]. After a new system of revenue-generation for hospitals had been introduced in China, over-prescription of drugs by doctors was observed: *“All these changes have induced many detrimental changes in medical care, such as irrational prescription of drugs for profits by doctors”* [44].

Similar problems in over-prescription of drugs were observed in Vietnam (within the medium HDI group), raising strong ethical concerns:

*This system of health care finances encourages over-prescription, especially the prescription of more expensive items. According to a recent study involving 27 private and non-private physicians in Ho Chi Minh City, current prescription practice is characterized as excessive, inappropriate, and “unethical”* [45]*.*

In countries that fall within the low and medium HDI categories, the lack of financial autonomy from political influence and international agencies was described as exacerbating the challenges raised by service modernization initiatives. For instance, the lack of donors’ long-term commitment limited the scaling up of new mental health services in Kenya: *“There are limited resources in the public sector and a lack of donor commitment to sustain large-scale implementation”* [46]. A similar lack of financial autonomy was described in Somalia:

*Because of a largely nonexistent tax base, the current system is highly donor dependent which means the system is totally vulnerable. This sentiment was also echoed by development partners who also cited low government expenditure and largely ear-marked donor funding as key financial challenges* [47]*.*

As mentioned earlier, accessibility to services is the most often examined health service delivery challenge and distinctions between its financial, sociocultural and geographical dimensions reveal challenges that countries may share across HDI categories. To begin with, affordability of services was scrutinized in rich countries that lack universal coverage, such as the U.S.:

*Although financial barriers to care are the most important in keeping individuals and families from seeking necessary health care, there are other barriers also in the way and underutilization of care by patients who delay or forego necessary care because of financial or other barriers* [48]*.*

In certain rich countries, limited geographical accessibility was described as resulting from the paucity of health centers in remote regions and the difficulties experienced by local communities in accessing them. This challenge was described in Canada:

*The geographic isolation and remoteness of [the First Nations] communities from larger urban centres has contributed to restricted access to health care and support services, and to the lack of services available in their communities* [49]*.*

Compromised geographical accessibility of services was also reported in poor countries where large water surfaces are found, like in the Pacific Islands [37], or where large deserts are found, like in Mongolia:

*Several factors particularly impede herder families’ ability to access basic health care services and life-saving medical treatment, including long distances to health services and lack of transportation; lack of money for health insurance; run-down hospitals, unqualified health personnel and lack of medical equipment; and high rates of referrals from primary to secondary/tertiary level of health care, which, in turn, are often inaccessible due to transportation and financial barriers* [50]*.*

Finally, acceptability of services has been described in very high HDI countries where sociocultural tensions in terms of ethnicity and gender arise. For instance, in the U.S.:

*The racial composition of the Mississippi physician population is not reflective of the racial composition of the patient population. This is a concern because patients report that they are most comfortable with a physician of their own race. A lack of diversity is also seen in the Mississippi physician human resources in respect to gender. While 25% of the nation’s physicians are female, one-half of that percentage (12–13%) is female in Mississippi* [51]*.*

#### Human resource challenges along the HDI

In proportion to the number of challenges reported, attention given to human resources challenges is much more important in low HDI countries than in very high HDI (ranging from 30% to 18%). This may be partly explained by the fact that poorer countries have fewer resources to allocate to medical and allied health professional schools, thereby lacking a continued and strong supply of human resources.

In low HDI countries, the lack of specialized staff in certain health sectors may affect the country as a whole, thus affecting an entire population of patients. For instance, in Ethiopia *“the scarcity of specialist mental health workers to support the delivery of mental health care by [primary health care] workers was evident across all sites”* [52]. More generally, the lack of human resources was linked to the exodus of the health workforce to the richest countries, a phenomenon that affects sub-Saharan African countries:

*The absence of an adequately trained health-care human resources and the so-called brain drain of substantial numbers of trained professionals out of Africa is a major problem for cancer control in sub-Saharan Africa* [32]*.*

The brain drain phenomenon is not, however, exclusive to low HDI countries; health workforce movements were described in countries falling within the high HDI category like Malaysia, where doctors shift from the public sector to the private sector, thereby inducing inefficiencies in the public sector.

*The growth of the private health sector has triggered the steady migration of senior doctors, specialists and experienced allied health professionals from the public sector to the more lucrative private healthcare sector* [53]*.*

In very high HDI countries, scarcity in human resources may affect a specific target population. In Canada, for example, Habjan and colleagues observed that First Nations communities were particularly affected by staff shortages:

*The concern about the shortage of local personal support workers was expressed in every community […]. A number of study respondents commented on the community’s needs for increased, reliable professional health services. In particular, they noted a deficiency in home nursing services, with nursing care available only during regular working hours and not on the weekends. In some communities, the community health nurse might come once or twice a week, but only if she is not detained in some other community* [49]*.*

Okamura reported the difficulty of striking the right balance between the number of health care providers and the number of beds and patients in Japan:

*The first [issue that Japan has to face] is the small number of doctors and nurses per hospital bed as well as population. This is attributable to the fact that compared with Europe and the U.S., Japan has a larger number of beds per population but fewer doctors and nurses* per capita [22]*.*

Finally, ensuring the competency of primary health care providers is intimately linked with the number of specialists available. This phenomenon may imply, in low HDI countries, that primary care providers could be performing tasks for which they are not adequately prepared:

*The human resource problems appear to be exacerbated by the fact that the bulk of staff working in psychiatric units are not mental health professionals by training. It was emphasized that although there are exceptions, the majority of mental health workers are general health care nurses and medical practitioners. Professionals working in the psychiatric units are largely untrained in even basic mental health care* [54]*.*

By contrast, in countries that belong to the very high HDI category, an inadequate ratio of primary care providers to medical specialists may entail competency and safety issues that are spread across the levels of care. For instance, in geriatric care in the U.S.:

*A patient may be taking drugs that either offset or potentiate one another in deleterious ways. Problems of this sort are compounded when the initial medical diagnosis is inaccurate, an event made more likely when physicians have had no exposure to geriatrics during medical school training* [55]*.*

Overall, our scoping review indicates that several categories of HS challenge are reported in roughly similar proportions across the four HDI categories: population and context, principles and values, leadership and governance, infrastructure and supplies, knowledge and information, and finances. While health service delivery challenges are more frequently reported in the articles examining HSs in very high HDI countries, human resources challenges are more frequently reported in the articles examining HSs in low HDI countries.

## Discussion

This scoping review relied on the dynamic HS framework developed by van Olmen and colleagues [7] and the HDI in order to identify key lessons from the available body of knowledge as well as knowledge gaps that call for further research. We address below these two contributions of our scoping review.

### Four key lessons

The first lesson that can be drawn from our scoping review is that the body of knowledge on HSs that has been produced since 2000 is not dominated by empirical research: only 33.6% of the articles that met our inclusion criteria were empirical studies and 37.7% were analyses that did not rely on primary research. This observation may be partly explained by the frequent use of administrative databases that contribute important information (e.g., number of beds, number of providers, population censuses, etc.) for government officials and health care managers. Yet, the reliance on such databases is less conducive to the production of cumulative empirical knowledge in health services research [56]. Increasing the production and publication of empirical studies on HSs is likely to require concerted action with health research funding agencies. Our findings also reflect the fact that empirical research tends to focus on specific interventions or programs, rather than examining a HS as a whole. This is unfortunate considering the importance of increasing our understanding of the ways in which the components of HSs dynamically interact.

Secondly, our study highlights that most of the available evidence on HS challenges relates to countries within the very high HDI category, including a large part of it that is specific to HSs in the U.S. Although this over-representation may be partly explained by a stronger support of HS research in rich countries and a publication bias that favours research conducted in these countries, the policy-oriented recommendations stemming from these studies may not be applicable to many other countries. Moreover, the majority of the articles we identified did not focus on a specific health sector and reported challenges for the population in general. The challenges reported are therefore described at macro level and the actors who are supposed to be acting upon such challenges remain unclearly defined. Our findings also showed that when specific sectors or populations were reported, the most frequently researched sectors included mental health, infectious diseases, primary care and non-communicable diseases. These trends may not only reflect the importance of these sectors in terms of burden of disease, but may also be associated to the development of sector-specific scientific journals. The most frequently studied target populations included elderly people, people living in remote or poor areas, visible or ethnic minorities, and children and adolescents. From a population health perspective, these groups are typically considered vulnerable and thus deserve careful attention by researchers. Yet, it is worth underscoring that the challenges that we extracted from the literature may be perceived differently by patients, health care providers and decision makers and it thus remains important to consider from whose perspective these challenges matter.

Thirdly, our findings underscore that the most frequently examined challenges since 2000 fall in three categories: health service delivery, human resources, and leadership and governance. This finding confirms that research on HSs has focused its attention on the supply, delivery and management of services, neglecting its initial intent to adopt an holistic perspective on health [1]. This may be due to deep-rooted disciplinary traditions and a poor responsiveness to the lessons learned by the broader health research community. Research themes such as governance and leadership and financing have indeed traditionally focused on issues closely related to the delivery of care and services. These themes could be addressed more holistically through research on community health partnership, for example, which would prove more aligned with a growing interest in the broader determinants of health. Although the WHO’s definition of HSs includes social, economic and environmental determinants, our findings indicate that the scientific literature on HSs challenges has focused on internal HS factors and less often examined how HSs address the broader determinants of health and integrate preventive interventions.

Fourthly, our scoping review shows that across the four HDI categories a roughly comparable level of attention has been given to six types of challenge: population and context, principles and values, leadership and governance, infrastructure and supplies, knowledge and information, and finances. Our findings also indicate that more attention has been given to health service delivery challenges in very high HDI countries than in low HDI countries. In contrast, more attention has been given to human resource challenges in low HDI countries than in very high HDI countries. Although our scoping review methodology cannot explain why exactly this is the case, it provided qualitative illustrations of the dynamic and complex relationships [57] that HS building blocks entertain. Structured around van Olmen and colleagues’ framework, our results showed that the adoption of new drugs and technologies, which fall in the infrastructure and supplies category, exacerbate the challenges already affecting human resources and finances. While this observation is not entirely new [58], our scoping review indicates that similar tensions are found across countries that belong to different HDI categories. For instance, our findings showed that waste of resources and overuse of services were reported in countries all along the HDI. As recently underscored by Brownlee et al. [59], the financial effect of an overuse of resources is well known in high HDI, but it is important to acknowledge that the spread of new technologies and drugs in low and middle HDI countries creates similar challenges. There is thus an opportunity to increase knowledge transfer and exchange among researchers who study HSs in a broad range of countries. Scientific journals may also be encouraged to consider their editorial policies along this line of reasoning.

### Three knowledge gaps

Despite the call for increased HS responsiveness to shifting demographics and health needs [1], our scoping review shows that not much research has examined the challenges falling within the population and context category. Nevertheless, these challenges often lie upstream of all the other categories of challenge [60]. For instance, a growing and more diversified population that is afflicted with a complex mix of chronic and acute diseases clearly affects how resources (human, material, informational, financial) should be deployed, managed and eventually reallocated. The body of HS literature that we analyzed thus focuses on challenges that arise as a consequence of shifts in population and context that may remain poorly understood. Further research should thus pay attention to these upstream dynamics, seeking to elicit the contextual changes that HSs should be responsive to. Paying due attention to population and context could also lead to revisit some of the “old” challenges like governance and leadership, financing and allocation of resources with a new lens.

Considering, on the one hand, the growing concerns about the financial sustainability of HSs and, on the other hand, the continued emergence since the turn of 2000 of new drugs and technologies, it is surprising that not much research has examined challenges specifically related to finances and to infrastructure and supplies. Perhaps further research could examine more specifically countries that fall within the high HDI category where challenges related to finances and new technology are currently fueling each other. Up to 49% of the articles we analyzed within this category referred to China, Malaysia and Brazil, which had an important economic growth in the last 20 years, and are likely to play an increasing role in the development and commercialization of health innovation in the near future [58].

Finally, in a context where evidence-based management and policy are increasingly touted [61], one may wonder whether challenges associated to the category knowledge and information have received sufficient attention. Important investments in knowledge and information have been made in countries all along the HDI, but a better understanding of the extent to which they enable HSs to thrive would prove very valuable [62].

### Strengths and limitations of our scoping review

The contribution of our scoping review to current knowledge should be interpreted considering its strengths and limitations. First, our inclusion criteria led us to ignore all the grey literature that is published by governmental agencies and consultants, which have accumulated important stocks of knowledge on regional, provincial and national HSs. Our decision to focus exclusively on the scientific literature was justified in view of the likely abundance of grey literature worldwide and our intent not to exclude a priori any country. Second, although we used in a consistent manner the definitions provided by van Olmen and colleagues and WHO [1, 7, 9, 14] to classify the challenges reported in the articles, we also sought to remain close to the authors’ own definitions. Yet, this implied that we had at times to make a final decision that would best serve the objectives of the scoping review. For instance, what some authors defined essentially as a health service financial accessibility challenge could be more fully articulated by others as an equity challenge, thereby falling into the values and principles category. Third, the distinction made by authors between a challenge and a solution was sometimes subtle. If, for example, a lack of motivation and incentives may constitute a challenge, a call to develop stronger incentives or increase salaries belong to the “solution” realm. Our analytical strategy was therefore to fully consider the context of the scientific article in which the challenges or the solutions were mentioned in order to remain consistent. Finally, this scoping review identified the challenges that have been studied by investigators and that met the editorial and scientific requirements of peer-reviewed journals. Greater prevalence in the scientific literature does not mean that the challenges reported are more acute or important from a policy perspective, but it stresses that these challenges are prevalent in the scholarship.

Among its strengths, our scoping review is, to our knowledge, the first study to use an explicit framework to classify all the HS challenges that had been documented worldwide since 2000. It also illustrated with several examples the relationships the various categories of challenge entertain among them. Another original aspect of our scoping review is to have clustered countries along the HDI, which is more compatible with the widely-shared aim of improving population health and wellbeing through HS strengthening. Building on the methodology of our scoping review, one could use the Gini coefficient to examine more specifically whether the reporting of HS challenges varies according to inequality.

## Conclusion

In the past decades, health services researchers and policymakers have increasingly been concerned by how HSs can respond to shifting health needs and adopt technological innovations [58]. Although this scholarship has sought to inform how certain types of technology such as computerized medical records or telehealth systems could strengthen specific HSs, very few studies sought to clarify, globally, the system-level needs and challenges that innovations should address in the first place. Within this perspective, we performed this scoping review as part of a broader research program in which we are examining how Responsible Innovation in Health (RIH) may help to reduce rather than exacerbate key HS challenges. Our premise is that the financing, development and commercialization of new technologies should be geared at supporting sustainable HSs. As a first step, this scoping review: 1) took stock of the scientific literature published since 2000 on HS challenges; 2) identified where this research was conducted, in which health sectors and on which populations; 3) examined the types of challenge that have been documented the most; and 4) explored whether the reported challenges varied across countries. Ultimately, a stronger command of HS needs and challenges should help to orient the work of those engaged in the development of health innovations.

## Additional file

Additional file 1: Search strategy (DOCX 21 kb)

## Abbreviations

HDI: Human development index
HS: Health system
U.S.: United States
WHO: World health organization

## Footnotes

1 We initially thought of classifying HSs and considered various taxonomies to this end. This option was discarded because of our intent to include articles that examined HSs in low- and middle-income countries and very few taxonomies can accommodate such diverse HSs. Furthermore, HSs may shift over time and we were reviewing the literature published since 2000.

2 Levac et al. [12.] offer a clear step-wise approach to scoping reviews, which builds on and complements the framework developed 5 years earlier by Arksey and O’Malley [11]. Levac et al.’s approach is more operational and provides useful lessons.

3 The articles that were excluded because of this criterion were written in Portuguese (n = 5), Spanish (n = 5) and Romanian (n = 1).
