# Supplementary material for: What do we know about the needs and challenges of health systems? A scoping review of the international literature
Source: BMC Health Serv Res. 2017 Sep 8;17:636. doi: 10.1186/s12913-017-2585-5 (PMC5591541; doi:10.1186/s12913-017-2585-5)
Supplement: Additional file 1: — Search strategy (DOCX 21 kb) [file 12913_2017_2585_MOESM1_ESM.docx]

**Appendix 1: Search strategy**

**Pub med:**

| **Search** | **Query** | **Items found** |
| --- | --- | --- |
| #2 | Healthcare system*[TI] OR Care system*[TI] OR Health system*[TI] OR  Delivery of healthcare[TI] or Delivery of health care[TI] OR Delivery of care[TI] OR Healthcare delivery[TI] OR Care delivery[TI] | 17599 |
| #3 | Delivery of health care[MAJR:NOEXP] | 44653 |
| #4 | #2 OR #3 | 56843 |
| #5 | Challeng*[TI] OR Need[TI] OR Needs[TI] OR Priorit*[TI] OR Reform*[TI] | 180050 |
| #6 | Health Services Needs and Demand[MAJR:NOEXP] OR Needs | 50955 |
| #7 | #5 OR #6 | 207201 |
| #8 | ((Health care[TI] OR Healthcare[TI]) AND Challeng*[TI]) | 1661 |
| #9 | ((#4 AND #7) OR #8) | 7604 |
| # 10 | #9 AND HASABSTRACT | 3482 |
| # 11 | #10 AND 2000:2016[DP] | 2603 |
| # 12 | #11 AND (ENG[LA] OR FRE[LA] OR ITA[LA]) | **2290** |

**Embase:**

| **Search** | **Query** | **Items found** |
| --- | --- | --- |
| 1 | ((Healthcare or Health or Care) adj2 (delivery or system$)).ti. | 27128 |
| 2 | *Health care system/ or *Health care delivery/ | 66332 |
| 3 | 1 or 2 | 83725 |
| 4 | (Challeng$ or Need or Needs or Priorit$ or Reform$).ti. | 210633 |
| 5 | *Needs Assessment/ | 4965 |
| 6 | 4 or 5 | 212839 |
| 7 | ((Health care or Healthcare) and Challeng$).ti. | 1787 |
| 8 | (3 and 6) or 7 | 7350 |
| 9 | limit 8 to abstracts | 3445 |
| 10 | limit 9 to yr="2000 -Current" | 2564 |
| 11 | 10 and (English or French or Italian).lg. | **2311** |

**PsycInfo:**

| **Search** | **Query** | **Items found** |
| --- | --- | --- |
| 1 | Title: ((Healthcare OR Care OR Health) NEAR/2 (delivery OR system*)) *AND* Year: 2000 *To* 2016 *AND* Peer-Reviewed Journals only | 4883 |
| 2 | Index Terms: "Health Care Delivery" *AND* Year: 2000 *To* 2016 *AND* Peer-Reviewed Journals only | 9914 |
| 3 | Index Terms:("Health Care Delivery") AND Year:[2000 To 2016]) *OR* (Title:(((Healthcare OR Care OR Health) NEAR/2 (delivery OR system*))) AND Year:[2000 To 2016]) *AND* Peer-Reviewed Journals only | 14,098 |
| 4 | Title: Challeng* OR Need OR Needs OR Priorit* OR Reform* *AND* Year: 2000 *To* 2016 *AND* Peer-Reviewed Journals only | 31,315 |
| 5 | Index Terms: "Health Service Needs" *OR* Index Terms: "Needs Assessment" *OR* Index Terms: "Health Care Reform" *AND* Year: 2000 *To* 2016 *AND* Peer-Reviewed Journals only | 6565 |
| 6 | (Index Terms:("Health Service Needs") OR Index Terms: ("Needs Assessment") OR Index Terms:("Health Care Reform") AND Year:[2000 To 2016]) *OR* (Title:(Challeng*  OR Need OR Needs OR Priorit* OR Reform*) AND Year:[2000 To 2016]) *AND* Peer-Reviewed Journals only | 35,822 |
| 7 | ((Index Terms:("Health Service Needs") OR Index Terms: ("Needs Assessment") OR Index Terms:("Health Care Reform") AND Year:[2000 To 2016]) OR (Title:(Challeng* OR Need OR Needs OR Priorit* OR Reform*) AND Year:[2000 To 2016])) *AND* ((Index Terms:("Health Care Delivery") AND Year:[2000 To 2016]) OR (Title: (((Healthcare OR Care OR Health) NEAR/2 (delivery OR system*))) AND Year:[2000 To 2016])) *AND* Peer-Reviewed Journals only | 1394 |
| 8 | Title: (("Health care" OR Healthcare) AND Challeng*) *AND* Year: 2000 *To* 2016 *AND* Peer-Reviewed Journals only | 223 |
| 9 | (Title:((("Health care" OR Healthcare) AND Challeng*)) AND Year:[2000 To 2016]) *OR* (((Index Terms:("Health Service Needs") OR Index Terms:("Needs Assessment") OR Index Terms:("Health Care Reform") AND Year:[2000 To 2016]) OR (Title:(Challeng* OR Need OR Needs OR Priorit* OR Reform*) AND Year:[2000 To 2016])) AND ((Index Terms:("Health Care Delivery") AND Year:[2000 To 2016]) OR (Title:(((Healthcare OR Care OR Health) NEAR/2 (delivery OR system*))) AND Year:[2000 To 2016]))) *AND* Peer-Reviewed Journals only | 1551 |
| 10 | Any Field: lang=(English OR French OR Italian) | 3,805,588 |
| 11 | Any Field:(lang:(English OR French OR Italian))) *AND* ((Title:((("Health care" OR Healthcare) AND Challeng*)) AND Year:[2000 To 2016]) OR (((Index Terms:("Health Service Needs") OR Index Terms:("Needs Assessment") OR Index Terms:("Health Care Reform") AND Year:[2000 To 2016]) OR (Title:(Challeng* OR Need OR Needs OR Priorit* OR Reform*) AND Year:[2000 To 2016])) AND ((Index Terms:("Health Care Delivery") AND Year:[2000 To 2016]) OR (Title:(((Healthcare OR Care OR Health) NEAR/2 (delivery OR system*))) AND Year:[2000 To 2016])))) *AND* Peer-Reviewed Journals only | **1506** |

**IBSS (Proquest):**

| **Search** | **Query** | **Items found** |
| --- | --- | --- |
| 1 | ((TI,SU((Healthcare OR Care OR Health) NEAR/2 (delivery OR system*)) AND TI,SU(Challeng* OR Need OR Needs OR Priorit* OR Reform*)) OR TI((Healthcare OR "Health care") AND Challeng*)) AND LA(English OR French OR Italian) Limits applied  Database :  International Bibliography of the Social Sciences (IBSS)  Limited to :  Date: From 2000 to 2016  Peer reviewed | **127** |

**Sociological abstracts (Proquest):**

| **Search** | **Query** | **Items found** |
| --- | --- | --- |
| 1 | ((TI,SU((Healthcare OR Care OR Health) NEAR/2 (delivery OR system*)) AND TI,SU(Challeng* OR Need OR Needs OR Priorit* OR Reform*)) OR TI((Healthcare OR "Health care") AND Challeng*)) AND LA(English OR French OR Italian) Limits applied  Database :  Sociological Abstracts  Limited to :  Date: From 2000 to 2016  Peer reviewed | **99** |

**Worldwide Political science abstracts (Proquest):**

| **Search** | **Query** | **Items found** |
| --- | --- | --- |
| 1 | ((TI,SU((Healthcare OR Care OR Health) NEAR/2 (delivery OR system*)) AND TI,SU(Challeng* OR Need OR Needs OR Priorit* OR Reform*)) OR TI((Healthcare OR "Health care") AND Challeng*)) AND LA(English OR French OR Italian) Limits applied  Database :  Worldwide Political Science Abstracts  Limited to :  Date: From 2000 to 2016  Peer reviewed | **66** |

**PAIS INTERNATIONAL (Proquest):**

| **Search** | **Query** | **Items found** |
| --- | --- | --- |
| 1 | ((TI,SU((Healthcare OR Care OR Health) NEAR/2 (delivery OR system*)) AND  TI,SU(Challeng* OR Need OR Needs OR Priorit* OR Reform*)) OR TI((Healthcare  OR "Health care") AND Challeng*)) AND LA(English OR French OR  Italian) Limits applied  Database :  PAIS International  Limited to :  Date: From 2000 to 2016  Peer reviewed | **103** |

**Web of science:**

| **Search** | **Query** | **Items found** |
| --- | --- | --- |
| #1 | TI=((Healthcare OR Care OR Health) NEAR/2 (delivery OR system*))  Indexes=SCI-EXPANDED, SSCI Timespan=All years | 22026 |
| #2 | TI=(Challeng* OR Need OR Needs OR Priorit* OR Reform*)  Indexes=SCI-EXPANDED, SSCI Timespan=All years | 325534 |
| #3 | #2 AND #1  Indexes=SCI-EXPANDED, SSCI Timespan=All years | 1505 |
| #4 | TI=(("Health care" OR Healthcare) AND Challeng*)  Indexes=SCI-EXPANDED, SSCI Timespan=All years | 1148 |
| #5 | #4 OR #3  Indexes=SCI-EXPANDED, SSCI Timespan=All years | 2478 |
| #6 | #5 AND PY=2000-2016  Indexes=SCI-EXPANDED, SSCI Timespan=All years | 1803 |
| #7 | (#5 AND PY=2000-2016) AND LANGUAGE: (English OR French OR Italian)  Indexes=SCI-EXPANDED, SSCI Timespan=All years | **1674** |
